# Batch and Continuous Ultrasound Assisted Extraction of Boldo Leaves (*Peumus boldus* Mol.)

**DOI:** 10.3390/ijms14035750

**Published:** 2013-03-12

**Authors:** Loïc Petigny, Sandrine Périno-Issartier, Joël Wajsman, Farid Chemat

**Affiliations:** 1 Université d’Avignon et des Pays de Vaucluse, INRA, UMR408, Avignon 84000, France; E-Mails: loic.petigny@univ-avignon.fr (L.P.); farid.chemat@univ-avignon.fr (F.C.); 2 BASF Beauty Care Solutions France, Lyon 69007, France; E-Mail: joel.wajsman@basf.com

**Keywords:** boldo, ultrasound, extraction, boldine, green extraction

## Abstract

Vegetal extracts are widely used as primary ingredients for various products from creams to perfumes in the pharmaceutical, nutraceutic and cosmetic industries. Having concentrated and active extract is essential, as the process must extract as much soluble material as possible in a minimum time, using the least possible volume of solvent. The boldo leaves extract is of great interest for the industry as it holds a great anti-oxidant activity due to high levels of flavonoids and alkaloids such as boldine. Ultrasound Assisted Extraction (UAE) has been used to improve the efficiency of the plant extraction, reducing extraction time, increasing the concentration of the extract with the same amount of solvent and plant material. After a preliminary study, a response surface method has been used to optimize the extraction of soluble material from the plant. The results provided by the statistical analysis revealed that the optimized conditions were: sonication power 23 W/cm^2^ for 40 min and a temperature of 36 °C. The optimized parameters of the UAE provide a better extraction compared to a conventional maceration in terms of process time (30 min instead of 120 min), higher yield, more energy saving, cleanliness, safety and product quality.

## 1. Introduction

Boldo (*Peumus boldus* Molina) is a plant naturally found in various climates from the warm and arid to the cold and rainy area of Chile [1]. It is widely used in various domains, from culinary preparation to medicines and is cited in several official pharmacopeias. Boldo leaves infusion is known for the digestives and hepato-biliairy protective effects [2]. Boldo leaves have a content of 0.4% of essential oil containing ascaridol, *p*-cymene, limonene [3]. Active compounds in boldo include alkaloids, flavonoids and other compounds. Among them, boldine ((*S*)-2,9-dihydroxy-1,10-dimethoxiaporphine) and catechin ((*2S*,*3R*)-2-(3,4-dihydroxy-phenyl)-3,4-dihydro-1(2H)-benzopyran-3,5,7-triol) are the main components of the alkaloid and flavonoid fractions in boldo leaves respectively. They are strong antioxidant agents [4,5]. Boldine undergoes peroxidative free-radical-mediated damage and acts as an efficient hydroxyl radical scavenger [6]. The extraction technologies are a keystone in cosmetics, pharmaceutics and nutraceutics but often requires up to 50% of investments in a new plant and more than 70% of total process energy [6]. As the cosmetic industry is one of the first to turn itself toward green chemistry, it is natural to think that it must work on the extraction process and aim toward the decrease of energy engaged. It must decrease the use and find alternative, recyclable and safer solvents. By reducing the overall toxicity of the process and final products as well as offer a safer process we can protect producer and consumer. By combining these objectives, it is possible to reach sustainability, therefore making extractions compliant with green chemistry. Then, we can therefore define: “Green extraction is based on the discovery and design of extraction processes which will reduce energy consumption, allow the use of alternative solvents and renewable natural products, and ensure a safe and high quality extract/product”. This definition helps to design six focuses for green extractions: vegetal matrix, solvent used, energy consumed, by-products created, process designed and the final extract [7]. Then, we can create new or adapt conventional extraction techniques and be able to reduce the impact of industry on our environment. It is possible through green-extraction to launch new innovative and environmental friendly cosmetics products. Many different kind of extraction exists and are widely used in cosmetics too.

Among these methods, we find Ultrasound Assisted Extraction (UAE). This technique is fast, consumes less fossil energy and permits the reduction of solvents, thus resulting in a purer product and higher yields. This method has been applied to extract food components such as aromas [8,9], antioxidants [10,11], pigments [12,13] and other organic and mineral components from a variety of matrixes. Ultrasound plays an important role as real potential sustainable technique for industrial applications for polyphenols extraction [14]. The cavitation process that occurs during sonication causes the rupture of cell walls, consequently enhancing solvent contact with available extractable cell material [15]. Using this capacity of ultrasound to abrade the vegetal matrix could be useful in the cosmetic industry, as vegetals extracts are widely used as primary ingredients in cosmetic formulas. The aim of this present work was to use UAE applied to boldo leaves for the extraction of soluble material and the evaluation of its effects on the yield and speed of extraction. A multivariate study was used to investigate the performance of the UAE as energy assistance in soluble material extraction from boldo leaves in term of yield, extraction time and to study the relevance of factors required during operating extraction. Optimal parameters were then obtained from a second-order polynomial equation. The optimal results were compared to those obtained using conventional maceration. As Boldine is an important compound of the plant responsible for a significant part of its anti-oxidative activity, HPTLC analysis at key time of conventional and ultrasound assisted extractions are conducted. The boldine analysis will serve as a model for interest extracted molecules and their possible degradation. This study will aim to assess the evolution and feasibility of a method of extraction from a batch “conventional” method of extraction by maceration to a batch Ultrasound Assisted Extraction and finally turned into a continuous mode of ultrasound assisted extraction.

## 2. Results and Discussion

### 2.1. Preliminary Study

**Figure 1:**
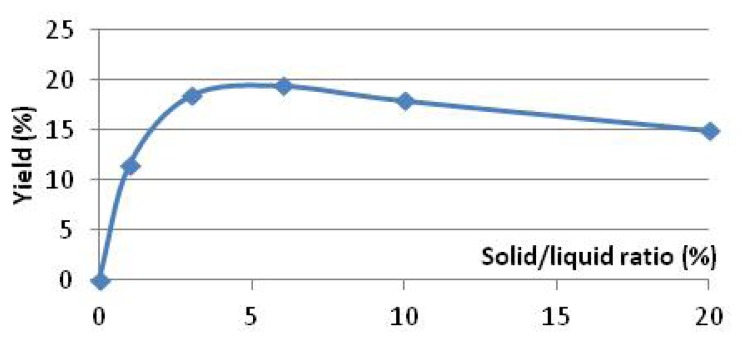
Yield of soluble material extracted in function of the S/L ratio.

To determine the optimum solid/liquid (S/L) ratio, the maximum yield of extraction was considered. It was observed that starting at 10% solid/liquid, boldo leaves tend to cluster together. In addition, a maximum yield was also observed for a ratio of 6% solid/liquid (Figure 1). The decreased yield can be explained by the increasing part of water absorbed in the rehydrated leaves, which represent the mass of dry leaves. Only 800 mL of extract in the experiment at 20% of S/L ratio was recovered were 1 L of water was engaged with 200 g of boldo leaves. These boldo leaves were weighted at 400 g at the end of the experiment. This loss of extract trapped in the matrix explains the decrease in yield for higher S/L ratio. The low yield obtained at 1% S/L could be explained by the large experimental error due to a low concentration of extract.

### 2.2. Experimental Design Studies

Three variables that could potentially affect extraction efficiency of soluble material were chosen: namely, the ultrasonic intensity, temperature and sonication time. These key-variables were involved in a central composite design in order to evaluate, optimize and conduct relevant ultrasound-assisted extraction of soluble material contained in boldo leaves. Ultrasonic intensity ranges from 9.9 W/cm^2^ to 23.1 W/cm^2^ (function of surface of sonotrode and applied ultrasonic power). Appropriate temperature setting is necessary in order to avoid destruction of organic compounds and/or permit future applications in pilot scale and also for efficient application of ultrasound (ultrasound effects are known to decrease with high temperatures). Thus, a moderate temperature range was chosen (9.7 ≤ *T* (°C) ≤ 70.0). Finally, we aimed to carry out extractions in a relatively short time, competitive with conventional procedure and interesting for future industrial applications. Thereby, sonication duration of experiments varied from 9.9 min to 40 min. These three controlled variables were studied in a multivariate study with 20 experiments (Table 1).

#### 2.2.1. Results for Soluble Material Extraction

Fully coded experiments and responses obtained for each run of the central composite design are presented on Table 2. As can be seen, responses varied widely function of parameters settings of experiments (from 16.5% to 23.8% of yield). Significance and suitability of the design were then studied using a variance analysis. Statistical significance of each effect (including interaction terms, linear and quadratic effects) was tested by comparing the mean squared against an estimate of the experimental error. Depending upon the degree of freedom (*Df.*) involved, *F*-ratio can be calculated (ratio of the mean squared error to the pure error). With a confidence level of 95%, *F*-ratio significance can be evaluated using the *p*-value column. In this column, when the value is lower than 0.05, the effect is significant (significant effects have been typed in bold). Four effects were found significant at a 95% confidence level in the experimental domain studied.

**Figure 2:**
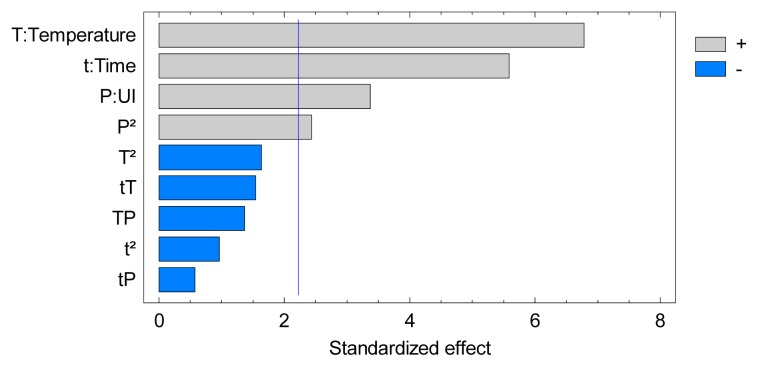
Standardized Pareto chart of optimization multivariate study.

This observation can also be pointed out on a Pareto chart of standardized effects, presented on Figure 2. Effects have been standardized and are proposed with a relative importance order. The lengths of bars are proportional to the absolute magnitude of the estimated effects coefficients while the vertical line represents the minimum magnitude of statistically significant effects for the response studied, with a 95% confidence interval. Linear effects of the three key variables (*P*, *T*, *t*) appear to be highly significant followed by the quadratic effect of the ultrasonic intensity (*P*^2^). The lack of significance of the cross-product terms (*P.T*, *P.t*, *T.t*) suggests the absence of interactions between variables.

The experimental data obtained from the CCD allowed us to determine an empirical relationship linking response studied (Yield) and key variables involved in the model (in coded units). Thus, a 2^nd^ order polynomial equation was obtained (1):

(1) $$\begin{array}{c} Y=8.50797+0.445797\times t+0.325932\times T-0.58338\times P-0.00252464\times t^{2}-\\ 0.00270062\times t\times T-0.0046296\times t\times P-0.00106796\times T^{2}-\\ 0.00551994\times T\times P+0.0335581\times P^{2} \end{array}$$

where, *Y* represents % of yield, *P* represents the applied density of power, *t* is the sonication time and *T*, the temperature in coded units. More than 90% of the variability of responses was explained (*R*^2^ statistics > 0.91), asserting a good accuracy and ability of the established model. *R*^2^*_adj_* is a regression coefficient adjusted for the number of coefficient involved in the model; it permits comparison between models with different numbers of independent variables and allows testing the goodness-of-fit of regression coefficient. Its value (>0.83) was also high, indicating a high degree of correlation between observed and predicted data.

#### 2.2.2. Optimization for Yield of Extraction

**Figure 3:**
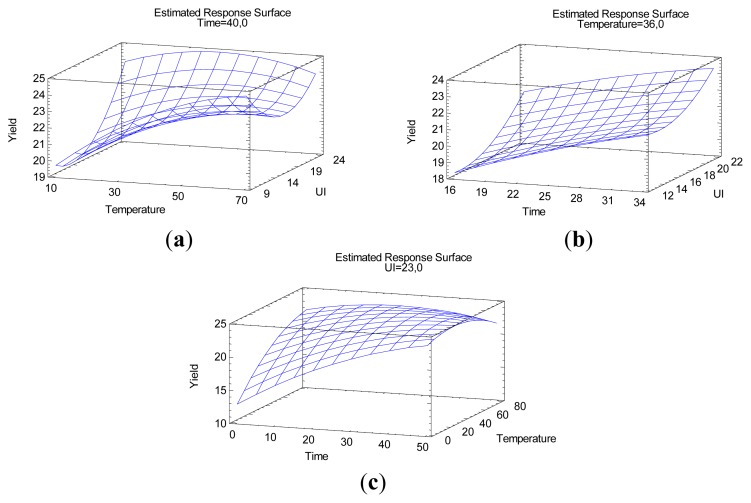
Optimization of ultrasound-assisted boldo leaves extraction by water: Yield (%) investigation in the multivariate study (**a**) Yield as a function of ultrasonic intensity (W/cm^2^) and Temperature (°C), (**b**) Yield as a function of ultrasonic intensity and sonication time (min), and (**c**) Yield as a function of temperature and sonication time.

A graphical representation can be introduced in order to visualize the significant relationship linking levels of variables and response studied (yield). The Figure 3 depicts three-dimensional plots, each plot highlighting the response behavior function of two variables with the third variable fixed to its optimum. The most influent variables are the linear terms of sonication duration (*t*) and temperature (*T*): yield increases linearly as sonication time and temperature increase. The same effect has been noticed with ultrasonic intensity (*P*) but with a less predominant influence as observed in the Pareto chart (Figure 2). A slight influence of quadratic effect of ultrasonic intensity (*P*^2^) is also illustrated on these surfaces (presence of surface curvature when ultrasonic intensity increases). Optimum Yield appears for the highest levels of variables. Optimal settings for yield maximization can be found solving equations system presented hereinafter (2):

(2) $$\frac{\delta (Y)}{\delta (P)}=0,\frac{\delta (Y)}{\delta (T)}=0\;and\;\frac{\delta (Y)}{\delta (t)}=0$$

Coded values obtained from these equations were then decoded and optimal settings were checked as follows: 23 W/cm^2^ for ultrasonic intensity, 36 °C for temperature and 40 min for sonication duration.

### 2.3. Comparison Study between Ultrasound Assisted Extraction and Conventional Maceration

**Figure 4:**
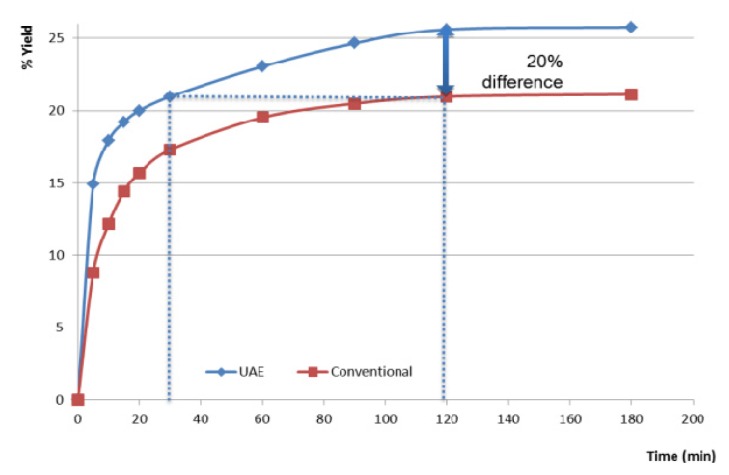
Comparison between conventional and UAE.

To evaluate the impact of ultrasound-assisted extraction in optimized conditions obtained from the response surface method, a comparison study was carried out between ultrasound and conventional maceration. From Figure 4, we can observe that ultrasound assisted extraction increased in yield by more than 20%. The comparison shows a clear improvement of the extraction, which is attributed to ultrasonic cavitation, since this is the only variable of treatment that differs in both experiments.

We can see that from 5 to 30 min of sonication, the yield is equivalent to the yield of conventional maceration at 15 to 90 min: UAE requires a third of the time to extract the soluble material of the leaves in conventional maceration. As we can see in Table 3, after 30 min of sonication, the dry extract ratio surpasses the one at 120 min of maceration, suggesting that the abrading of the leaves by sonic cavitation frees deeper soluble material unreachable through conventional maceration. Also, at similar yield and unfiltered, the extract from UAE is much more cloudy and green than the extract from maceration which is browner and clear suggesting more plant particles in suspension after UAE. After 0.45 μm filtration, both extract of the same dry extract ratio are undistinguishable. A HPTLC test is done at 30 and 120 min for both conventional maceration and UAE extracts to further the comparison with boldine as standard.

**Figure 5:**
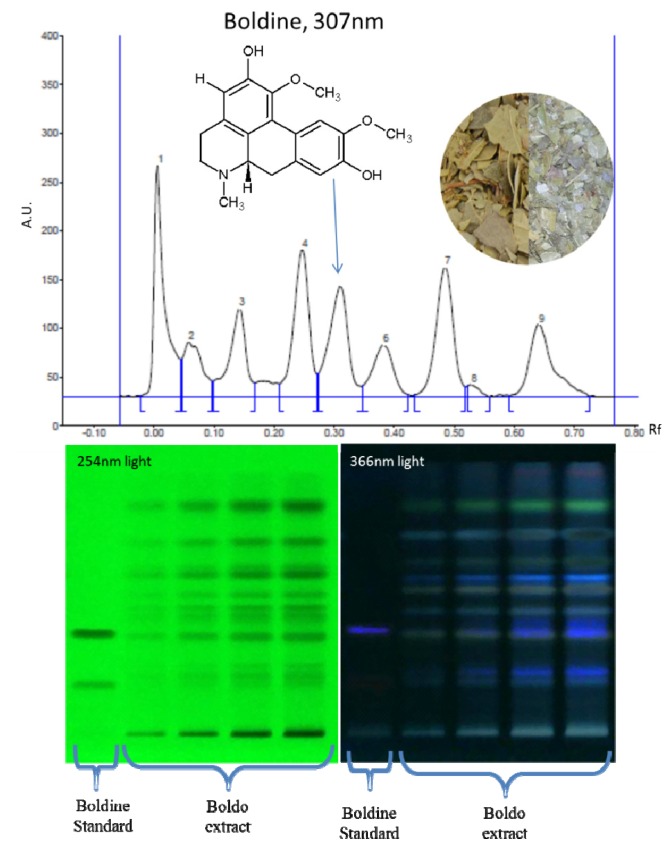
HPTLC chromatogram of hydrolyzed extract and boldine standard at 254 nm and 366 nm and the densitogram of a hydrolyzed extract of boldo track at 307 nm.

A typical plate of HPTLC boldo extract analysis of boldine can be seen in Figure 5, along with an absorbance chromatogram at 307 nm of a track where boldo extract were sprayed. The densitometric analysis and integration of the identified peaks of boldine allowed us to quantify the amount of boldine in the samples. As showed in Table 3, the quantity of boldine extracted per gram of boldo leaves with 30 min of sonication is equivalent to 2 h of conventional maceration and twice as much as 30 min of maceration. In addition, after 2 h of sonication, the amount of boldine extracted is 50% higher than 2 h of maceration. The increase of boldine extraction following the same kind of trend as total soluble material extraction implies that the process accelerate effectively the extraction, even for key molecules. In addition, it did not show any clues of degradation.

**Figure 6:**
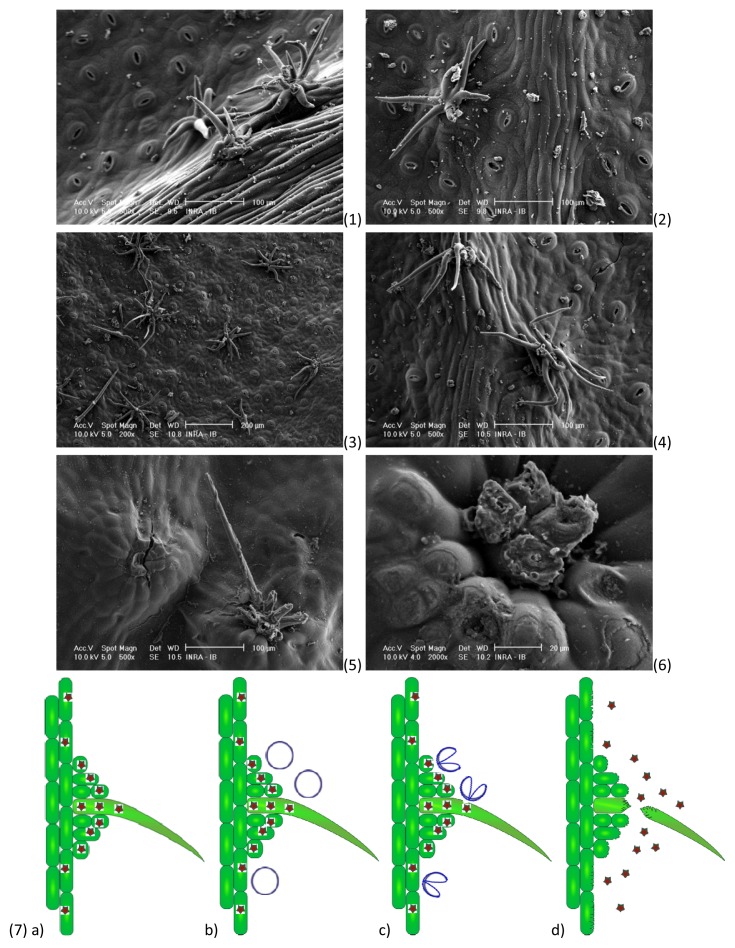
(**1**,**2**) Boldo leaf, magnification 500 on the original picture scale; (**3**,**4**) Boldo leaf after 2 h of maceration, magnification 200 and 500, respectively, on the original picture scale; (**5**,**6**) Boldo leaf after UAE at optimized conditions, magnification 500 and 2000 respectively; on the original picture scale; (**7**) Representation of a cavitation bubble collapsing on the surface of the boldo leaf. (**a**) Plant profile with a trichome at the surface of the leaf, (**b**) Generation of a cavitation bubble, (**c**) Collapse of the cavitation bubble which generates a micro-jet directed toward the surface and (**d**) Abrasion of the surface, breaking of the trichome, and release of soluble material in the surrounding medium.

Boldo leaves issued from both conventional and ultrasonic extractions process are observed and compared with unprocessed boldo leaves by SEM. After 2 h of maceration, the most fragile structures of the leaf only start to show damage on the “hair” like structure of the trichomes (Figure 6(3–4)), but most of them are undamaged as they are on unprocessed leaves (Figure 6(1–2)). It is however not the case after ultrasonic treatment: most “hair” structures of the trichome are cut and broken. If one remains, it is heavily damaged (Figure 6(5)). The cells around the structures also appear to have suffered heavy abrading (Figure 6(6)). One way to explain the difference between the results of extraction in UAE and conventional maceration could be based on the contact surface between the leaf and the water. In conventional maceration, the water is in contact with the leaf on both side, but also on its perimeter. The perimeter per gram of leaves can be increased by crushing more the leaves. In UAE, the cavitation abrades all the surface of the leaves (Figure 6(a–d)). Then, the surface of contact between leaf and water increases over time and give a deeper access and direct contact with water inside the leaf, where conventional maceration must rely on diffusion of the soluble material through the leaf.

### 2.4. Ultrasounds Effects on Extracted Molecules

In order to verify if the UAE is safe for the extracted molecules, an aqueous solution of boldine is submitted to the optimized ultrasound extraction conditions. The degradation of boldine is quantified by comparing the mass before and after ultrasound treatment using HPTLC densitometric analysis. The boldine is recovered at 98.66% after ultrasound treatment, which is within 3% experimental error.

### 2.5. From Batch to Continuous System of Extraction

The interest of UAE lies in the reduced cost due to decrease in time of extraction, a more effective and focused use of power, a better yield and more concentrated extract at comparable S/L ratio. Conventional maceration is time and energy consuming and generally not always interesting from industrial point of view. On the many advantages the UAE brings as a process, we can highlight that no other chemical than water is used for the extraction. In addition, as the extraction takes a reasonable amount of time (30 min) it is possible to imagine a conversion of the batch system toward a continuous system. An experimental “pilot” study was carried out in a continuous piston apparatus. From the previous lab study, the selected conditions for the ultrasound extraction pilot study were at the optimum conditions of temperature (36 °C) and of ultrasound density of power (23 W/cm^2^). In this study, 30 min are required to perform an effective and relevant extraction of soluble material similar to a conventional maceration; therefore, this time of extraction is chosen rather than 40 min calculated by optimization. Although, the yield of soluble material from the ultrasound extraction pilot device was equal to batch UAE at equal time of extraction (21.7% yield of extraction). The result showed that the potential use of ultrasound extraction was promising for extraction on an industrial scale by using the flexibility of the method

## 3. Experimental Section

### 3.1. Plant Material and Chemicals

Boldo leaves collected in May 2011; contain 5% of moisture. Boldo leaves are crushed 5 s with a blender before use (Robot Coupe Blixer 2, Montceau-en-Bourgogne, France). As solvents, Chloroform HiPerSolv chromanorm, diethylamine GPR rectapur, chlorhydric acid 35% rectapur come from VWR Prolabo (Darmstadt, Germany). The toluene analytical reagent is provided by Fisher (Illkirch, France). The methanol HPLC for Gradient Analysis is provided by Acros Organics (Slangerup, Denmark). Ammonia puriss is bought to Sigma Aldrich (St. Louis, MO, USA). The standard boldine for HPTLC and degradation study comes from Extrasynthèse (Genay, France).

### 3.2. Extraction Procedures

Preliminary studies were performed allowing us to determine an optimal S/L ratio for the rest of the investigations. Mixture of water and boldo leaves was stirred; rotation of the stirrer is set up at 250 rpm in order to keep an efficient mixing of all leaves without the creation of vortex.

**Figure 7:**
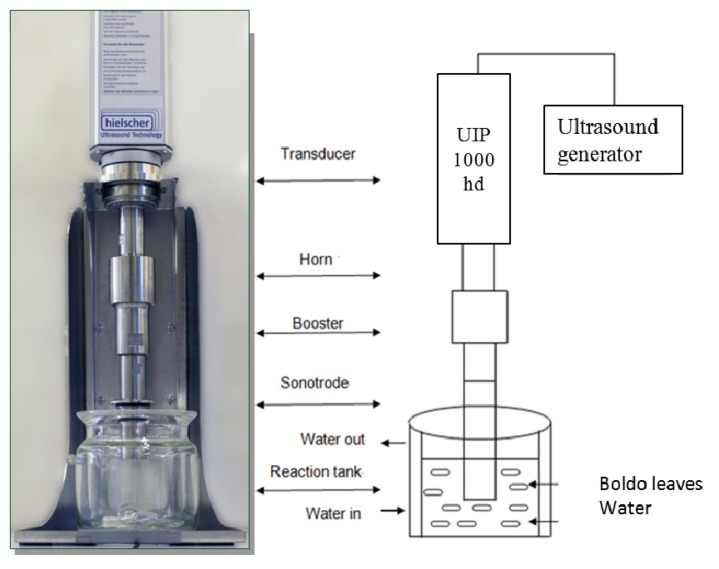
Batch ultrasonic assisted extraction.

Ultrasound-assisted extraction experiments were performed with a sonotrode (BS2d34, Hielscher UIP 1000 hd, [16]) and a glass reaction tank [17]. The double-layered mantle of the reactor (allows the control of extraction temperature by a cooling system by water circulation. The transducer is connected to the horn with a “booster” installed in amplification mode and finally the sonotrode, which is immersed to the middle of the liquid and boldo leaves filling the tank (Figure 7). For a typical extraction procedure, samples of boldo leaves were extracted with 1 L of distillated water at atmospheric pressure. Time of extraction ranges between 10 and 40 min, with an Ultrasonic Intensity of 10 to 23 W/cm^2^, and a temperature range of 10 to 70 °C.

Conventional maceration, made for comparison, was carried out in the exactly same conditions of time, temperature and S/L ratio, with a stirrer instead of ultrasound.

**Figure 8:**
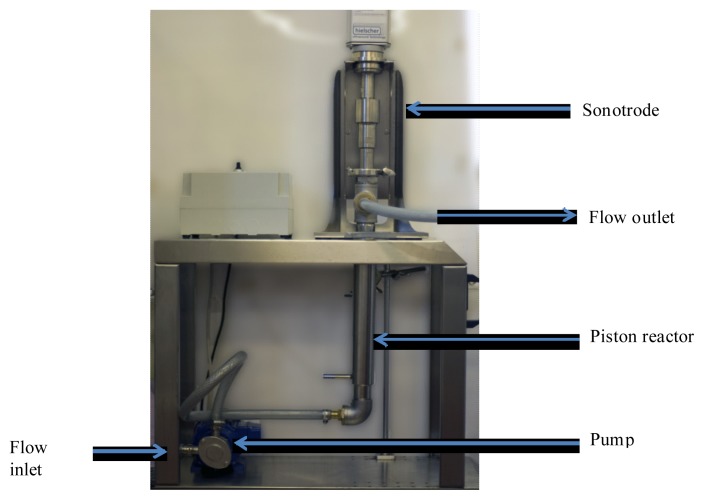
Ultrasonic pilot continuous.

Continuous ultrasound assisted extraction is carried out with an apparatus (Figure 8) made of a circulatory pump (M71p4 Caparelli RHEUS). The inlet is in a large beaker containing water and boldo leaves. The pipes bring the flow from the inlet beaker to the pump, then to the sonication tube in ascending flow. The sonication tube is specially fitted to the sonotrode. Then, the flow comes out the sonication tube toward the outlet beaker.

To determine the yield of extraction, an aliquot of extract from conventional and UAE is centrifuged (Sigma 4–16K Centrifuge) at 3000*g* for 5 min and filtered (0.45 μm Millipore nitrocellulose filter) under 100 mbar vacuum to avoid unnecessary boiling at room temperature and thus, artificial concentration of the sample. This aliquot is analyzed on a moisture analyzer.

### 3.3. Yield of Extraction Determination

Yield of extraction is determined through the use of a moisture analyzer (OHAUS MB35). 5 g of 0.45 μm filtered sample are heated 45 min at 110 °C to obtain the mass stability. This method gives us the water content of the extract, therefore also the soluble material content. From this soluble material content, we can deduce the yield of soluble material extracted from the leaves (3).

(3) $$Yield=(100\%-\%\;water\;content\;of\;the\;extract)\times \frac{\text{Mass }of\;extract}{initial\;Mass\;of\;leaves}$$

### 3.4. Isolated Compound Study

In order to verify whether boldine in the extract undergoes degradation during the sonication, a solution of boldine is submitted to the ultrasound treatment. With a final concentration of 0.02 mg/mL, the solution is placed in the glass reactor, followed by an ultrasound treatment under the optimized conditions by the experimental design. One sample untreated and one sample treated by ultrasound was then analyzed by HPTLC for quantification. All analyses are carried out in triplicates.

### 3.5. Boldine Analysis

#### 3.5.1. Hydrolysis

5 g of the extract were acidified with 0.5 mL of hydrochloric acid (6 M, Rectapur, VWR Prolabo, Fontenay-sous-Bois, France) at 100 °C for 10 min. Then, after cooling, the mixture was basified with an ammonium solution of 1 mL at 25% (Puriss, Sigma Aldrich, St. Louis, MO, USA). The hydrolyzed sample was extracted with 5 mL of chloroform (HiPerSolv Chromanorm, VWR Prolabo, Fontenay-sous-Bois, France) and both phases are placed in a tube for centrifugation (at 1500*g* for 5 min). The organic phase was collected and dried at 45 °C under a nitrogen stream. The dry extract was solubilized with 5 mL of chloroform, filtered on 0.45 μm PTFE syringe filter put in vial for HPTLC analysis.

#### 3.5.2. HPTLC analysis

The samples and standard were spotted in the form of bands of 8mm width with a Camag microliter syringe controlled by the Automatic TLC Sampler ATS 4 (Camag, Muttenz, Switzerland) on precoated silica gel glass Plate 60 Å F254 (20 × 10 cm; Merck, Darmstadt, Germany). The plates were prewashed by propan-2-ol and activated at 120 °C for 20 min prior to spotting. A constant application rate of 200 nL/s was employed and space between two bands was 8.5 mm.

The migration of the plate was carried out in 20 × 10 cm twin glass chamber of an Automatic Development Chamber ADC2 (Camag, Muttenz, Switzerland). The plate is first dried for 5 min to evaporate any residual solvent from the samples and standards. The mobile phase consisted of toluene-methanol-diethylamine (40:5:5 *v*/*v*/*v*) and 10 mL of mobile phase were used per chromatography. The glass chamber was saturated for 10 min at 25 °C and at relative humidity of 45% with an additional 10 mL of mobile phase. The TLC plate is pre-saturated for 10 min. The length of chromatography run is of 60 mm to allow results in better apparent resolution, with more convenient capability of the detecting device to perform integration of peak area. Subsequent to the development, TLC plates were dried in a current of air from the ADC2.

The densitometric analysis was performed on CAMAG TLC Scanner 3. The slit dimension was kept 6.00 × 0.10 mm and 10 mm/s Scanning speed was employed. The scanning was performed at 307 nm in reflectance/absorbance mode. The source of radiation used was Deuterium/Tungsten lamp emitting a continuous UV spectrum between 190 and 400 nm. Each track is scanned and baseline correction is used. All operations were monitored by WinCATS software (V 1.4.7.2018, Camag, Muttenz, Switzerland). Standard of boldine were prepared by solubilizing 10 mg of boldine (Extrasynthèse, Genay, France) in 500 mL of chloroform. Five spots of the standard of increasing volume were made to obtain concentration rage of 50–300 ng/spot. The median concentration of the range is repeated 5 times on the TLC plate.

### 3.6. Experimental Design

A response surface methodology has been used to investigate and optimize extraction of soluble material from Boldo leaves. A Box-Wilson procedure, commonly called Central Composite Design (CCD), was used to evaluate the relevance of the three controlled factors in extraction process (namely ultrasonic intensity, temperature and sonication duration) and identify eventual interactions between variables. This multivariate study provides a complete exploration of the experimental domain studied using a two-level full factorial design (coded ± 1), superimposed by center points (coded 0) and “star points” (coded ± α), thus permitting a number of experiments shortened. “Star points” are axial experiments located on variables axes at a distance α from the center, thus establishing new extremes for the parameters of the factors involved. These extremes points provide estimation of the curvature for the model. Its value is function of properties desired for the design and depends upon the number of experiments involved in the model. A virtual cube where each axis corresponds to a variable can be devised in order to represent the multivariate study. In this study, the central composite design points describe a sphere around the factorial cube. Preliminary experiments allowed us to point the variables implied in the model at five separated coded levels: −α (= −1.68), −1, 0, +1, +α (= +1.68). Values are presented on Table 1 and involved a total of 20 experiments; including six replications at the center point to evaluate experimental error measurement, and randomized to avoid effects of extraneous variables. Variables were coded according to the following equation (4), where *X_i_* is the coded value, *x_i_*, the real value of a variable, *χ̄_i_*, the real value of a variable at the center point, and Δ*x_i_*, the step change:

(4) $$X_{i}=\frac{x_{i}-\bar{x_{i}}}{\Delta x_{i}}$$

After extraction and dry mass analyses, the yield of extraction is considered as response variable and studied using the CCD procedure. Various designs, models and interpretations were obtained and analyzed using statistical software: Statgraphics Plus 2000 (Manugistics, Rockville, MD, USA). Experimental data for predicting results have then been represented using a 2^nd^ order polynomial equation (5) as follows:

(5) $$Y=\beta_{0}+\sum_{i=1}^{n}\beta_{i}{\text{X}}_{i}+\sum_{i=1}^{n}\beta_{ii}{\text{X}}_{i}^{2}+\sum_{i=1}^{n-1}\sum_{\begin{array}{l} j=2\\ j>i \end{array}}^{n}\beta_{ij}{\text{X}}_{i}{\text{X}}_{j}$$

where, *Y* is the response variable that can be the Yield of extraction β_0_ is the average response obtained at replicated experiments of the CCD, β*_i_*, β*_ii_*, β*_ij_* are the linear, quadratic and cross-products effects, respectively, *X_i_* and *X_j_* are the independent coded variables.

### 3.7. Scanning Electron Microscopy

Three boldo leaves samples are made directly after the comparison experiments: one witness sample boldo leaves without treatment, another from the 2 h of maceration method and the last from UAE with the optimized conditions. They are directly golden plated before being observed by scanning electron microscopy, scans at 10 kV.

## 4. Conclusions

The proposed UAE procedure is less time-consuming and allows a rapid process of extraction for boldo leaves. A multivariate study allowed us to define optimal settings for a rapid extraction of soluble material assisted by ultrasound. The different investigations showed that high power ultrasound could enhance analyte release of vegetal matrix material at a better rate compared with conventional method: equal yield of extraction at 30 min with a fourth of the time required for conventional maceration. These lab scale results of the transition from batch to batch ultrasound and toward continuous ultrasound assisted extraction could lead toward industrial scale test and switch in process dynamic from batch to continuous. Investigations with an ultrasonic pilot continuous (Figure 8) device have been realized for industry tests and ultrasound appears to be a viable option for boldo leaves extract. This process can be considered as a sustainable alternative for the cosmetic industry since it allows simplified handling, time reduction, quantity of targeted extracts improved, and indicating the potential for the use of ultrasound extraction on an industrial scale.

## Figures and Tables

**Table 1:** Variables involved in the Central Composite Design (CCD) and response obtained.

Run	Sonication time (min)	Temperature (°C)	UI * (W/cm^2^)	Yield (%)
1	25.0	40.0	16.5	20.6
2	25.0	40.0	16.5	20.6
3	25.0	40.0	16.5	21.2
4	25.0	40.0	16.5	20.4
5	25.0	40.0	16.5	20.7
6	25.0	40.0	16.5	20.3
7	16.0	22.0	12.6	16.5
8	16.0	58.0	12.6	21.7
9	34.0	22.0	12.6	20.7
10	34.0	58.0	12.6	23.0
11	16.0	22.0	20.4	19.5
12	16.0	58.0	20.4	22.0
13	34.0	22.0	20.4	21.9
14	34.0	58.0	20.4	23.8
15	25.0	9.7	16.5	16.5
16	25.0	70.3	16.5	21.4
17	9.9	40.0	16.5	17.3
18	40.1	40.0	16.5	21.4
19	25.0	40.0	9.9	20.0
20	25.0	40.0	23.1	22.8

* UI: Ultrasound Intensity.

**Table 2:** ANOVA in the CCD.

Source	Sum of squares	*Df*	Mean square	*F*-Ratio	*p*-Value
A:Time	20.166	1	20.166	31.25	0.0002
B:Temperature	29.7028	1	29.7028	46.03	0
C:UI *	7.34062	1	7.34062	11.37	0.0071
AA	0.597778	1	0.597778	0.93	0.3585
AB	1.53125	1	1.53125	2.37	0.1545
AC	0.21125	1	0.21125	0.33	0.5799
BB	1.72989	1	1.72989	2.68	0.1326
BC	1.20125	1	1.20125	1.86	0.2024
CC	3.82983	1	3.82983	5.93	0.0351
Total error	6.45333	10	0.645333		
Total (corr.)	73.4655	19			

* UI = Ultrasound Intensity; *R*-squared = 0.912; *R*-squared (adjusted for d.f.) = 0.833.

**Table 3:** Summary and comparison of extractions.

Method of extraction	Time of extraction (min)	Yield of extraction (% of leaves boldo solubilized in the extract)	Boldine (μg of boldine/g of boldo leaves)
UAE	30	21.8	100
Conventional	30	18.0	51.7
UAE	120	26.7	148
Conventional	120	21.5	99.5
